# Dosimetric assessment of TomoDirect radiotherapy and TomoHelical radiotherapy in the clinical implementation of total skin irradiation

**DOI:** 10.3389/fonc.2025.1645834

**Published:** 2025-09-01

**Authors:** Haiyang Wang, Yifei Pi, Chunbo Liu, Panfeng Wang, Chengzhang Long, Yaping Qi, Fanyang Kong, Bin Han, Fangna Wang, Fei Jia, Lele Liu, Dandan Xu, Tengfei Ji, Huijuan Wu, Yuexin Guo

**Affiliations:** ^1^ Department of Radiation Oncology, The First Affiliated Hospital of Zhengzhou University, Zhengzhou, China; ^2^ Medical and Ionizing Radiation Metrology Testing Research Center, Henan Provincial Institute of Metrology and Testing Sciences, Zhengzhou, China; ^3^ Division of Ionizing Radiation Metrology, National Institute of Metrology, Beijing, China

**Keywords:** TomoDirect, TomoHelical, total skin irradiation, auxiliary ring structures, complete-block mode

## Abstract

**Purpose:**

This study aims to compare the technical characteristics of TomoDirect (TD) radiotherapy and TomoHelical (HT) radiotherapy in total skin irradiation (TSI) applications. We conducted a comprehensive evaluation of dosimetric parameters and delivery efficiency in TD-based treatment planning to establish clinical guidelines for modality selection in mycosis fungoides.

**Materials and methods:**

This retrospective study analyzed eight mycosis fungoides patients treated with TSI between June 2020 and June 2023, utilizing a 5-mm-thick diving suit bolus to enhance the skin dose distribution with a prescription of 24 Gy delivered in 20 fractions (five fractions/week). Thermoplastic masks (3 mm in thickness) were used for head/neck and thorax/abdomen region immobilization, while the lower limbs were immobilized in a vacuum cushion. Comparative treatment planning employed both TD and HT techniques, with TD plans utilizing 7, 9, and 11 equally spaced coplanar beams (0°starting angle). Ring0, Ring1, Ring2, Ring3, and Ring4, which were 1-cm thick away from the planning target volume (PTV) at distances of 0, 1, 2, 3, and 4 cm, and other normal tissues (NT) were generated. The auxiliary structures were completely blocked during planning. The other plan parameters remained consistent. Plan quality assessment compared the target mean dose (PTVmean), homogeneity index (HI), conformity index (CI), and organ-at-risk (OAR) doses between techniques, with additional evaluation of treatment delivery efficiency through time comparisons.

**Results:**

When using NT, Ring4, and Ring3 auxiliary structures in complete-block mode, TD plans with more than nine beams demonstrate comparable PTVmean, HI, and CI-to-HT plans, whereas TD plans of less than nine beams exhibit inferior target coverage. Neither HT nor TD plans meet the clinical requirements when Ring2, Ring1, or Ring0 structures are fully blocked. Regarding OAR sparing, nine-beam TD and HT plans show equivalent maximum doses to optical structures (lenses, optic nerves, and chiasm) and mean doses to other OARs (oral cavity, salivary glands, lungs, heart, liver, and kidneys) with NT/Ring4/Ring3 blocking. However, both techniques fail to satisfy the OAR constraints when Ring2/Ring1/Ring0 are blocked. Treatment delivery times remain similar between modalities with NT/Ring4/Ring3 blocking, but the efficiency significantly decreases for both when deeper structures (Ring2/Ring1/Ring0) are included in the blocking protocol.

**Conclusion:**

When employing complete-block mode for NT, Ring4, and Ring3 structures, TD plans utilizing more than nine beams demonstrate comparable dosimetric performance to HT plans in terms of target coverage, OAR sparing, and treatment delivery efficiency. However, both modalities fail to meet the clinical dosimetric requirements when deeper-seated structures (Ring2/Ring1/Ring0) are included in the blocking protocol.

## 1 Introduction

Cutaneous T-cell lymphoma (CTCL), a rare group of mature T-cell malignancies primarily involving the skin, accounts for approximately 71% of primary cutaneous lymphomas, with mycosis fungoides (MF) being the most prevalent subtype (MF) (1). According to the latest data from the National Cancer Institute’s “Surveillance Epidemiology and Outcomes”, CTCL (mainly MF) is currently growing at a rate of 9.6 cases/million per year, and the incidence rate accounts for approximately 50% of CTCL (2). CTCL is usually highly radiosensitive and has traditionally been treated with total skin electron irradiation (TSEI), which is also clinically considered one of the most effective methods for treating CTCL (3). The conventional Stanford six-field technique presents practical limitations due to extended treatment distances and required patient repositioning (4). With the continuous development of radiotherapy technology, especially with the emergence of HT (5), its unique 360° helical irradiation and pneumatic multileaf collimator can realize ultra-long target treatment (160 cm × 40 cm) and dose sculpture distribution, which is very suitable for the treatment of long and complex targets, such as multiple metastasis irradiation, cranio-spinal irradiation, total body irradiation, total bone marrow irradiation, etc. (6), and it also solves well the drawbacks existing in traditional TSEI. With the gradual development of HT technology, TD treatment technology has been added. Its fixed beam intensity modulation is similar to that of conventional accelerators, but it has the characteristics of up to 12 fixed beams. TD irradiation is intensity-modulated radiation therapy of HT (7), wherein the accelerator head remains fixed at a specific angle while the treatment couch moves along the head–foot direction of the target. This modulation adjusts the intensity of the radiation reaching the target and OARs by controlling the opening and closing MLC to meet clinical and OAR dose requirements. Lin et al. (8) were the first to conduct effectiveness tests on the dose buildup effect of neoprene wetsuits using anthropomorphic phantoms. Hsieh et al. (9) were the first to use a 3-mm diving suit as a bolus to achieve total skin helical tomotherapy (TSHT). Similarly, Haraldsson et al. (10) also used a diving suit as a bolus to perform TSHT, while Wang et al. (11) utilized a 3D-printed total skin bolus for the same purpose. Currently, there is no feasibility study on total skin irradiation using TD irradiation mode. This article will compare the dosimetric differences between TD treatment plans and HT treatment plans with different numbers of beams in TSI treatment by using different auxiliary ring structures to evaluate which plan can achieve better protection of OARs while ensuring target dose distribution, thereby providing more options for the clinical implementation of TSI technology.

## 2 Materials and methods

### 2.1 Patients’ clinical characteristics

Eight patients with mycosis fungoides underwent total skin irradiation (TSI) at the Radiation Therapy Department of the First Affiliated Hospital of Zhengzhou University between June 2020 and June 2023. The cohort comprised five male and three female patients (**Table 1**), with an age range of 35–70 years (mean: 59 years), height range of 150–178 cm (mean: 164.8 cm), and weight range of 40–95 kg (mean: 65.1 kg). All methods were carried out in accordance with relevant guidelines and regulations. All experimental protocols were approved by the Zhengzhou University Committee on Ethics Review of Life Sciences (approval number: 2024-KY-0076). Informed consent was obtained from all subjects and/or their legal guardian(s).

**Table 1:** Characteristics of the eight patients.

Patient no.	Age (years)	Sex	Diagnosis	Height (cm)	Body weight (kg)	Treatment technique
1	31	M	MF	170	95	TSHT
2	49	M	MF	178	78	TSHT
3	42	M	MF	165	65	TSHT
4	38	F	MF	150	55	TSHT
5	52	F	MF	160	51	TSHT
6	60	M	MF	168	63	TSHT
7	65	F	MF	155	40	TSHT
8	56	M	MF	172	74	TSHT

### 2.2 Bolus

Eight patients were dressed in a 5-mm diving suit bolus. The diving suits were tailored according to the patient’s external shape to achieve a tight wrap around the body.

### 2.3 Immobilization

Patients dressed in 5-mm diving suits were immobilized in supine position. Thermoplastic masks (3 mm in thickness) were used for head/neck and thorax/abdomen region immobilization, while the lower limbs were immobilized in a vacuum cushion, ensuring optimal patient positioning and dosimetric accuracy. The upper anatomical reference (“upper mark”) was placed at the umbilicus level, and the lower anatomical reference (“lower mark”) was positioned at the patella level. The segment line made of lead was located approximately 10 cm above the patella as the boundary between the upper and lower targets.

### 2.4 Image acquisition at simulation

Computed tomography (CT) scans (Brilliance CT Big Bore, Philips Healthcare, Cleveland, OH, USA) were performed under the following conditions: a scan and reconstruction slice thickness of 5 mm. The patients were scanned in the upper and lower segments—the upper segment was scanned from the skull vertex to 10 cm below the lead wire boundary, and the lower segment was scanned from the toes to 10 cm above the boundary. This 20-cm overlap region (10 cm above and below the wire) ensured proper dose blending between treatment segments while accounting for setup variations and beam penumbra.

### 2.5 Delineation of target volumes and organs at risk

The CT images were transferred to the eclipse 15.6 workstation (Varian Medical Systems, Inc. Palo Alto, CA, USA). The target volumes and OARs for all patients were delineated by radiation oncologists based on the planning CT according to the ICRU50 (12) and ICRU62 reports (13). The clinical target volume (CTV) was defined as the region between the skin surface and 5 mm beneath it (14). To account for setup errors and the dose buildup effect, the CTV was initially expanded uniformly by 5 mm to create a preliminary PTV. However, since this expansion could extend beyond the body contour (e.g., into air), the outer region of the PTV was retracted by 3 mm to ensure anatomically plausible boundaries while maintaining an adequate target coverage. This approach balanced the geometric uncertainties with physical feasibility, optimizing dose delivery to superficial tissues. OARs were delineated based on the ICRU 83 report (15), including the total bone marrow (head and neck bones, upper limb bones, ribs, spine, pelvis, lower limb bones), eyeballs, lens, parotid, lungs, heart, kidneys, liver, bladder, rectum, spinal cord, brainstem, etc. The junction between the upper and lower sections of the total body irradiation (TBI) had been studied in our previous publication (16). The dose in the overlap region was mostly homogeneous when the distance was equal to the FW.

### 2.6 Plan designs

**Figure 1:**
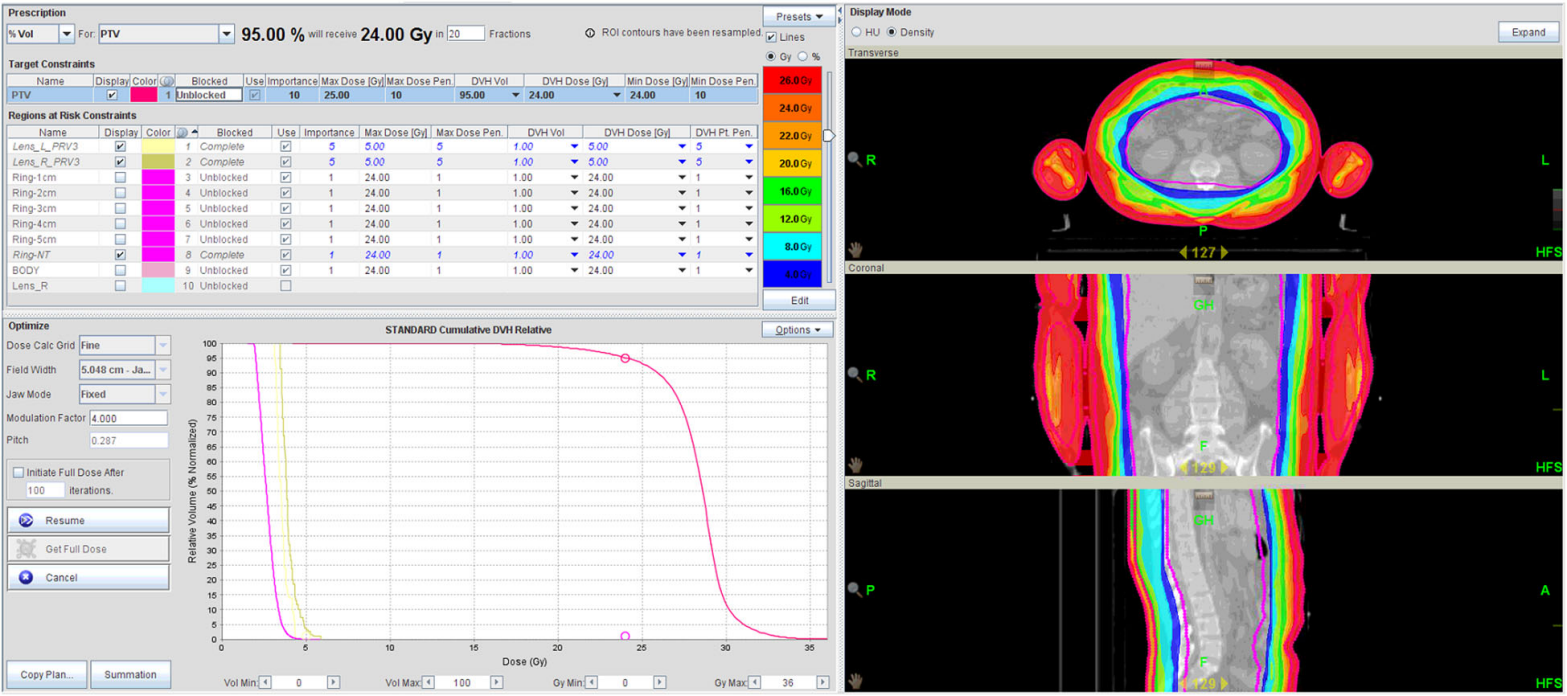
TSI-TD treatment plan for the patient.

The planned CT and contoured structures of each patient were transferred to the treatment planning workstation (version 5.1.6; Accuray, Sunnyvale, CA, USA) for preparation. The dose prescription was 24 Gy in 20 fractions (five fractions/week). The PTV gradually retracted from 1 to 5 cm by 1 cm step to create the ring-shape auxiliary structure as Ring0, Ring1, Ring2, Ring3, Ring4, and other normal tissues (NT). The auxiliary structures were used as an assistant tool for plan optimization to achieve dose objectives. During planning, all the auxiliary structures were set to complete mode one by one. Different number of beam plans for TD and HT were designed. The TD plans with 7, 9, and 11 equally spaced beams were created, starting at an angle of 0°. The planning required at least 95% of the target to receive the prescription dose, with FW of 5 and 2.5 cm, pitches of 0.287 and 0.215, and MF of 4 and 3. The dose grid was 0.195 cm × 0.195 cm (**Figure 1**). Plans were designed by combining different parameters and auxiliary structures. All other parameters remained consistent, and the final dose calculation was performed after 100 iterations for each plan.

### 2.7 Assessment of plan parameters

The parameters assessed for the patients included PTVmean, HI, and CI of the target volume. At least 95% of the target volumes reached the prescribed dose. HI was calculated using the formula, HI = D5%/D95%, where D5% is the dose received by 5% of the PTV volume, and D95% is the dose received by 95% of the PTV volume. An HI value greater than 1 represents the heterogeneity dose distribution of the target volume. CI was obtained using the following Paddick equation (17), CI = VT_,ref_/VT × VT_,ref_/V_ref_, where VT_,ref_ is the target volume covered by the prescription isodose (cm^3^), V_ref_ is the volume encompassed by the prescription isodose (cm^3^), and VT is the target volume (cm^3^). If the CI value is closer to 1, the better the dose conformity of the target volume is.

### 2.8 Statistical analysis

All statistical analyses were conducted using SPSS Statistics (version 19.0; IBM Corp., Armonk, NY), with continuous variables presented as mean ± standard deviation (mean ± SD). Graphical representations were generated using OriginPro (version 8.0; OriginLab Corp., Northampton, MA, USA).

## 3 Results

### 3.1 Comparisons of dosimetric parameters of target

**Figure 2:**
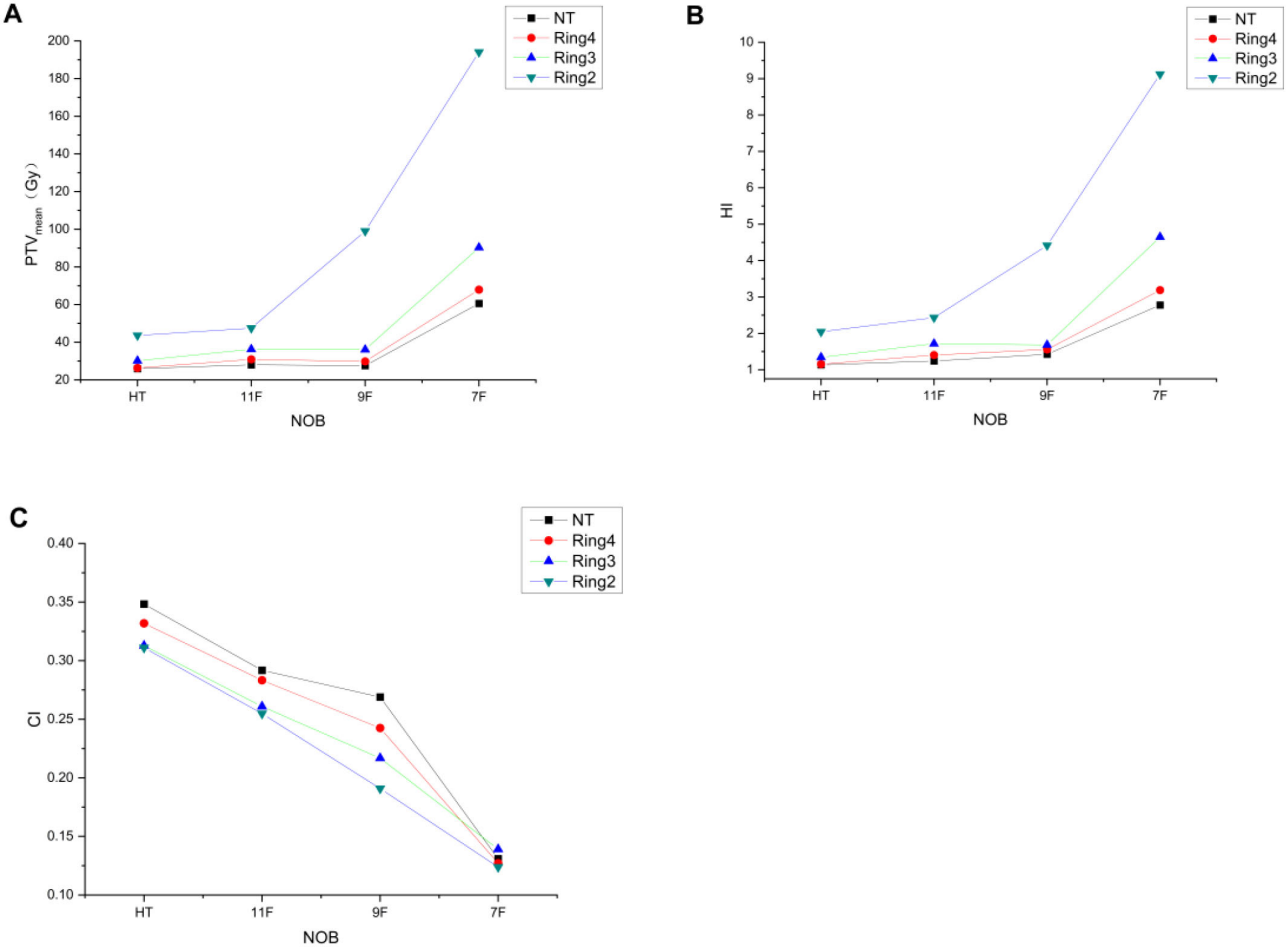
Trends of PTVmean **(A)**, HI **(B)**, and CI **(C)** variation with the decreased number of beams (NOB) and irradiation mode under different auxiliary structures.

**Figure 2A** demonstrates that TD plans utilizing 11 beams and 9 beams achieve prescription dose coverage equivalent to HT plans when NT, Ring4, and Ring3 structures are set to complete-block mode. At the same time, HI (**Figure 2B**) and CI (**Figure 2C**) are consistent with the abovementioned results. However, the TD plan with seven beams in the complete mode using the Ring2 auxiliary structure cannot achieve the same prescription dose as the HT plan and is also consistent with the results of HI and CI. This consistency aligns with previous research indicating a consistent relationship between auxiliary structures and target distance, ensuring that when the auxiliary structure distance from the target exceeds or equals 3 cm in the complete mode, the target dose meets the clinical requirements. Our analysis confirmed comparable target coverage between thermoplastic mask-immobilized regions (head/neck/thorax/abdomen) and diving-suit-only areas (lower limbs), with no statistically significant differences (**Table 2**) in PTVmean (24.1 ± 0.3 Gy vs. 23.9 ± 0.4 Gy, *p* = 0.15) or D95% coverage (96.2% ± 1.1% vs. 95.8% ± 1.3%, *p* = 0.22).

**Table 2:** Comparison of dosimetric parameters between thermoplastic mask regions and diving suit regions.

Dosimetric parameters	Thermoplastic masked regions	Diving suit-only regions	P
PTVmean	24.1 ± 0.3 Gy	23.9 ± 0.4 Gy	0.15
D95% coverage	96.2% ± 1.1%	95.8% ± 1.3%	0.22

### 3.2 Comparisons of dosimetric parameters of OARs

**Figure 3:**
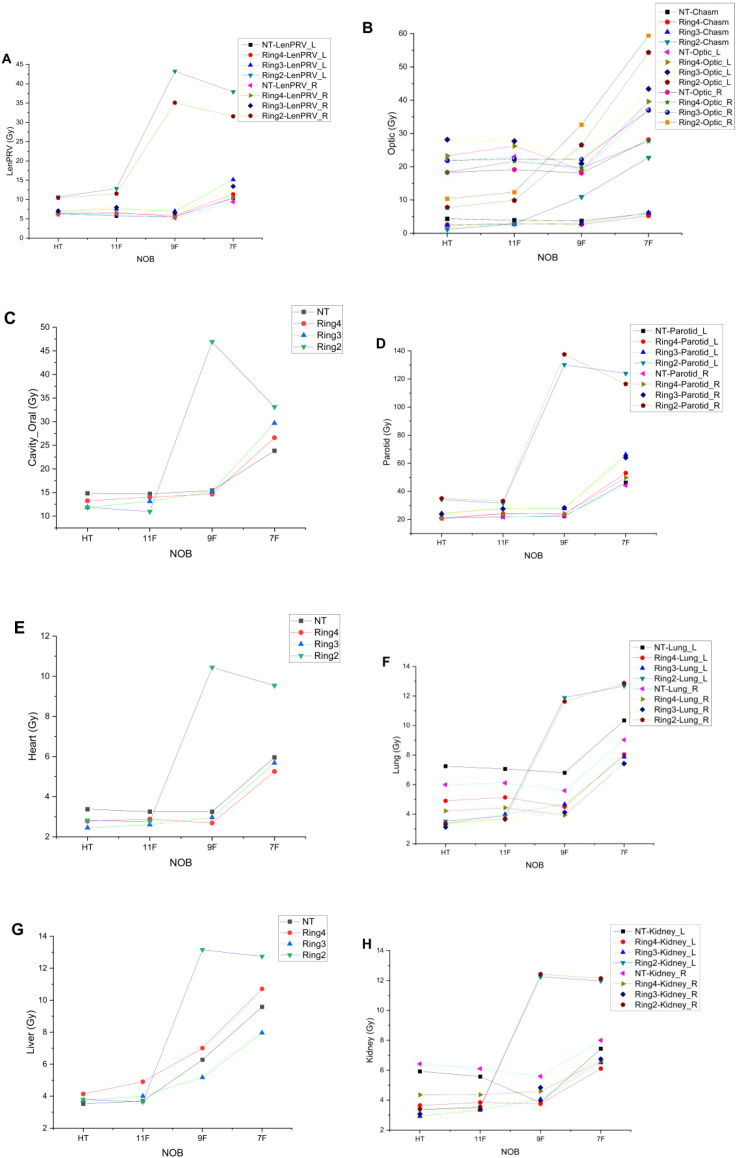
Trends of OAR variation with the decreased NOBs and irradiation mode under different auxiliary structures. LenPRV **(A)**, Optic **(B)**, Cavity_Oral **(C)**, Parotids **(D)**, Heart **(E)**, Lungs **(F)**, Liver **(G)** and Kidneys **(H)**.

**Figures 3A–H** show the maximum or average doses to the left and right lens, optic nerves, chiasm, oral cavity, bilateral parotid glands, lungs, heart, liver, and bilateral kidneys under the NT, Ring4, Ring3, and Ring2 auxiliary structures in the complete mode, with HT irradiation as well as with the 11-, 9-, and 7-beam TD plans. From the figures, it is apparent that to ensure that organ-at-risk doses remain within clinically acceptable ranges, auxiliary structures must be selected as NT, Ring4, or Ring3. Additionally, the 11- and 9-beam TD plans align with HT plans in terms of OAR doses. However, the Ring2 auxiliary structure and seven-beam TD plan fail to meet the clinical requirements.

### 3.3 Comparisons of beam on time and gantry period

**Figure 4:**
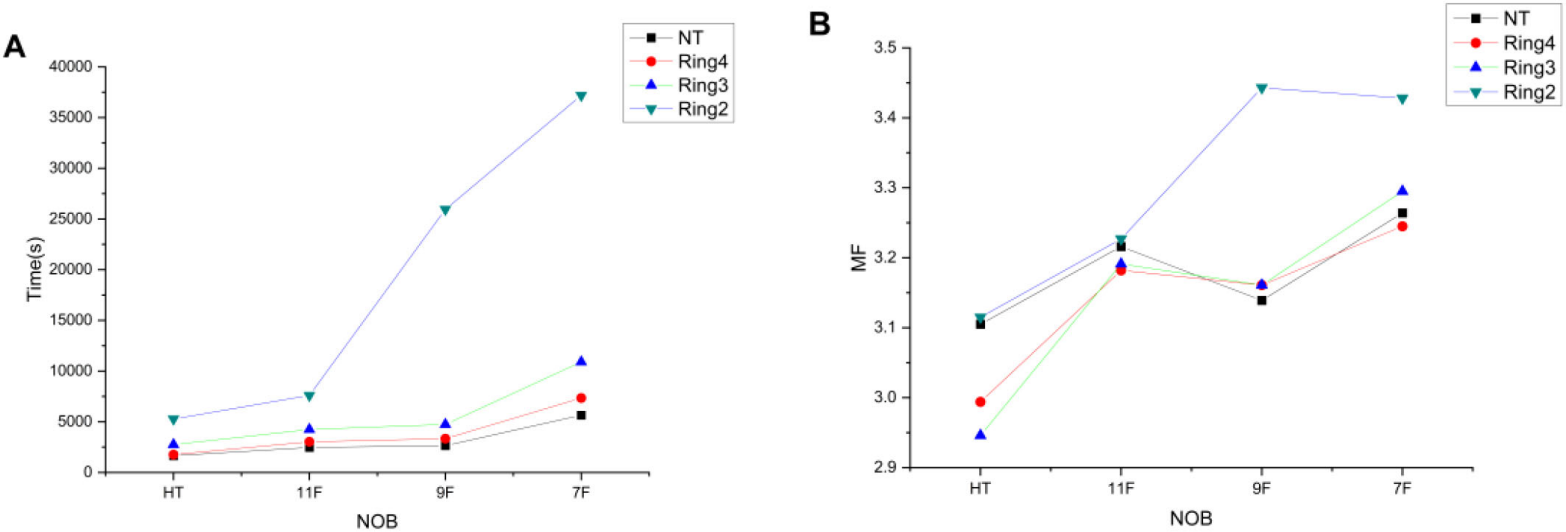
Trends of time **(A)** and MF **(B)** variations with the decreased NOBs and irradiation mode under different auxiliary structures.

In **Figure 4A**, when the auxiliary structures are NT, Ring4, and Ring3, there is little variation in the treatment delivery time between HT plans and TD plans with 11 and 9 beams, whereas the treatment delivery time significantly increases for the TD plan with seven beams. For the auxiliary structure Ring2, both HT plans and multi-beam TD plans show a significant increase in treatment delivery time, which can no longer meet the clinical treatment demands. In **Figure 4B**, he MF values of the TD plan are significantly higher than those of the HT plan, with no apparent pattern of variation.

In summary, during TSI treatment, conventional HT mode can be utilized along with TD plans featuring auxiliary structures such as NT, Ring4, and Ring3 with 9 or 11 beams, yielding consistent outcomes. This offers patients requiring TSI treatment a broader range of therapeutic options.

## 4 Discussion

TD irradiation technology is currently a hot topic in clinical treatment research and application. Primarily, its application in breast cancer irradiation has garnered significant attention. This includes studies on TD irradiation technology in the treatment of unilateral breast cancer (18, 19) as well as its application in the treatment of bilateral breast cancer (20, 21). Additionally, research has also explored its use in cranio-spinal irradiation treatments (22, 23), esophageal cancer therapy (24, 25), and TBI treatment (26, 27). TD irradiation technology is increasingly being applied clinically. However, there are currently no reports of clinical studies on the application of TD in TSI treatment.

Our team previously studied the relationship between different auxiliary structures and outcomes in TSI-HT technology, and we systematically investigated the effects by setting distances from the target at 0, 1, 2, 3, and 4 cm and the remaining volume to generate auxiliary structures Ring0, Ring1, Ring2, Ring3, Ring4, and NT with a uniform thickness of 1 cm each. The auxiliary structures were sequentially set to complete mode in the treatment plan design. The results revealed that when using auxiliary structures with a distance from the target greater than or equal to 3 cm and employing the complete mode, PTVmean, CI, and HI met the clinical requirements. As the distance between the auxiliary structures and the target increased gradually, the treatment delivery time decreased accordingly, but the volume of normal tissues receiving excessive radiation increased. Conversely, when the distance from the target to the auxiliary structures was less than 3 cm and the complete mode was applied, the clinical requirements could not be met (14). In this study, we focused on investigating the effects of 7, 9, and 11 equalized beams, considering the influence of auxiliary ring structures. We aimed to explore whether TD technology and HT technology have advantages in terms of radiation utilization and treatment delivery time, thereby providing more options for radiotherapy techniques in clinical TSI treatment.

The tomotherapy system has advanced to the fourth generation. The first generation lacks a fixed-beam irradiation mode, while fixed-beam irradiation functionality has been incorporated since the second generation. The functions that can be achieved are the same as those of conventional accelerators: three-dimensional conformal radiotherapy, intensity-modulated radiation therapy, and volumetric-modulated arc therapy. However, their unique attributes differentiate them from conventional accelerators in terms of application and clinical outcomes (7). The institution currently employs a second-generation HT model capable of implementing the aforementioned three treatment modalities. The research discussed in this article is applicable to the second, third, and fourth-generation models. However, since the first-generation HT does not have the described functions, the abovementioned research results are not applicable.

The TD treatment technique can accommodate up to 12 treatment beams (28), considering that even-numbered beams lead to intersecting beams, which are unfavorable for intensity modulation. Therefore, this study did not investigate them. The focus of this research was on odd-numbered equally spaced beams with fewer than 12 beams. There are reports that the starting angle has no effect on the results in TBI-TD research. Therefore, the starting angles of all fixed beams in this study all start from 0°.

All patients wore a 5-mm diving suit bolus. Unlike previous studies by Hsieh et al. (9), who used a 3-mm diving suit bolus, or Haraldsson et al. (10), who used a 7-mm diving suit bolus, we opted for the most common and widely available 5-mm diving suit bolus. This choice enhances the convenience of procurement and ensures the generalizability of the research findings. While different thicknesses of diving suits may yield minor variations in results, the overall impact is deemed insignificant, thus allowing for broader applicability of the conclusions. However, when considering different materials for a bolus, especially those with significant density variations, further research is needed to confirm conclusions.

The PTV gradually moves inward to form auxiliary structures, creating Ring0, Ring1, Ring2, Ring3, Ring4, and NT auxiliary structures with thicknesses of 1 cm each at distances of 0, 1, 2, 3, and 4 cm and the remaining volume, respectively (14). These auxiliary structures do not represent OARs or the target but are solely used as tools for plan optimization, enabling dose constraints within the body and serving as part of the plan evaluation to study the trend of auxiliary structure dose distribution with varying distances. Generated by moving the PTV inward, Ring0 through Ring4 and the NT auxiliary structure are created with a uniform thickness of 1 cm at specified distances from the target. These structures are generated according to specific requirements on the treatment planning system and remain unchanged due to human efforts, thus ensuring the universality and representativeness of the research findings and their clinical applicability.

This study generated a total of five rings: Ring0, Ring1, Ring2, Ring3, and Ring4 with a thickness of 1 cm and the remaining volume NT. The decision to generate only five rings instead of more auxiliary rings was primarily because the smallest cross-section of the head and neck region is typically approximately 10 cm, making it impractical to generate additional auxiliary rings. It is also noted that the longest distance auxiliary structure used in related research is 5 cm (29). The auxiliary ring structures selected in this study are all 1 cm in thickness, without generating thinner auxiliary rings (such as 8, 5, and 3 mm). The main consideration is that while thinner auxiliary structures might provide more detailed results compared to the 1-cm-thick ones, the changing trend of the research results should be consistent. Thus, this article did not conduct research on thinner auxiliary rings.

This study only collected data from Ring2, Ring3, Ring4, and NT, excluding data from Ring0 and Ring1. This omission is primarily due to the inability to optimize the treatment planning for Ring0 and Ring1 auxiliary structures when used in the complete mode, as their proximity to the target is too close. Therefore, statistical data for Ring0 and Ring1 are not available.

All patients used a 5-mm diving suit bolus. Currently, the most commonly used diving suit on the market is the 5-mm-thick one, which is readily available and offers good material uniformity. Therefore, this conclusion has broader applicability. For the use of other materials as a bolus, especially those with significant differences in density and thickness, this conclusion may not be applicable and requires further investigation.

Previous studies have employed varying bolus thicknesses for TSI treatment. Hsieh et al. (9) used a 3-mm diving suit, achieving an adequate skin dose but with potential underdosing in deeper subcutaneous tissues due to reduced bolus thickness. Haraldsson et al. (10) utilized a 7-mm diving suit, which improved the dose homogeneity but increased the scatter dose to normal tissues. Our study adopted a 5-mm diving suit as a balanced choice, ensuring reliable target coverage while minimizing excessive scatter. This thickness is widely available and clinically practical, with results showing comparable target coverage to HT plans (e.g., PTVmean within ±2% of prescription dose). Minor variations in bolus thickness (3–7 mm) did not significantly alter the clinical outcomes, supporting the generalizability of our protocol.

Our TD-based TSI protocol utilizing 9 or 11 beams with optimized auxiliary structures (Ring3/Ring4/NT) demonstrated clinically acceptable conformity (CI: 0.90 ± 0.03) and homogeneity (HI: 1.05 ± 0.02) indices comparable to HT while reducing the treatment time by 15%–20%. The strategy of positioning auxiliary structures ≥3 cm from the target effectively controlled high-dose spillage to normal tissues (V20Gy reduction, *p* < 0.05), validating the protocol’s efficiency and dosimetric reliability for clinical implementation.

The study systematically evaluated odd-numbered beam configurations (7/9/11) to avoid modulation challenges from beam intersections, establishing 9 and 11 beams as the optimal range for TD-based TSI. By standardizing the 5-mm bolus and auxiliary ring methodology (1-cm increments), we provide a reproducible framework that expands the treatment options, particularly for centers lacking tomotherapy capabilities. This approach not only maintains dosimetric quality but also improves operational efficiency, offering a viable alternative to HT with comparable clinical outcomes.

It is important to acknowledge several limitations in this study. Firstly and most significantly, the study lacks *in vivo* dosimetry verification. We did not use Thermoluminescent Dosimeters (TLDs), Optically Stimulated Luminescence Dosimeters (OSLDs), or films to measure the delivered surface dose, which is a critical component for validating any TSI protocol. While rigorous immobilization and daily Megavoltage Cone Beam Computed Tomography (MVCT) were employed to ensure geometric accuracy, these measures do not substitute for direct dose measurement. Secondly, a formal robustness analysis, which would involve evaluating the plan’s sensitivity to setup uncertainties and patient motion, was not performed. The primary scope of this work was to investigate the dosimetric feasibility of TD planning, and these validation steps were beyond that initial scope. Furthermore, our conclusions are drawn from a small patient cohort (*n* = 8), a limitation dictated by the rarity of mycosis fungoides requiring TSI and the preliminary nature of this technical investigation. These limitations together underscore that while our findings establish a promising planning methodology for TD-based TSI, further comprehensive validation—including phantom-based measurements, *in vivo* dosimetry, and robustness analysis—is essential before this technique can be broadly adopted in clinical practice.

While this study primarily focused on establishing the technical feasibility and dosimetric performance of TD-based TSI treatment, we fully agree that investigating patient-specific characteristics (e.g., anatomical variations, disease subtypes, or individual radiosensitivity) could yield valuable insights for personalized treatment optimization.

In conclusion, to achieve results comparable to HT technology in TSI treatment, TD plans with 9 or 11 beams can be utilized, along with auxiliary structures such as NT, Ring4, and Ring3. This study highlights the applicability of TD technology in TSI treatment, thereby offering a wider range of treatment options for TSI therapy.

## Data Availability

The original contributions presented in the study are included in the article/supplementary material. Further inquiries can be directed to the corresponding author.
